# Is accurate and reliable blood loss estimation the 'crucial step' in early detection of postpartum haemorrhage: an integrative review of the literature

**DOI:** 10.1186/s12884-015-0653-6

**Published:** 2015-09-28

**Authors:** Angela Hancock, Andrew D. Weeks, Dame Tina Lavender

**Affiliations:** School of Nursing, Midwifery & Social Work, The University of Manchester, Jean McFarlane Building, Oxford Road, Manchester, M13 9PL UK; Department of Women’s and Children’s Health, Liverpool Women’s Hospital, University of Liverpool, Crown Street, Liverpool, L8 7SS UK

**Keywords:** Blood loss estimation, Evaluation strategies, Postpartum haemorrhage, Recognition, Early diagnosis

## Abstract

**Background:**

Postpartum haemorrhage (PPH) is the leading cause of maternal mortality in low-income countries and severe maternal morbidity in many high-income countries. Poor outcomes following PPH are often attributed to delays in the recognition and treatment of PPH. Experts have suggested that improving the accuracy and reliability of blood loss estimation is the crucial step in preventing death and morbidity from PPH. However, there is little guidance on how this can be achieved. The aim of this integrative review was to evaluate the various methods of assessing maternal blood loss during childbirth.

**Methods:**

A systematic, integrative review of published research studies was conducted. All types of studies were included if they developed, tested, or aimed to improve methods and skills in quantifying blood loss during childbirth, or explored experiences of those involved in the process.

**Results:**

Thirty-six studies were included that evaluated the accuracy of visual estimation; tested methods to improve skills in measurement; examined their effect on PPH diagnosis and treatment, and / or explored additional factors associated with blood loss evaluation. The review found that health professionals were highly inaccurate at estimating blood loss as a volume. Training resulted in short term improvements in skills but these were not retained and did not improve clinical outcomes. Multi-faceted interventions changed some clinical practices but did not reduce the incidence of severe PPH or the timing of responses to excessive bleeding. Blood collection bags improved the accuracy of estimation but did not prevent delays or progression to severe PPH. Practitioners commonly used the nature and speed of blood flow, and the condition of the woman to indicate that the blood loss was abnormal.

**Conclusions:**

Early diagnosis of PPH should improve maternal outcomes, but there is little evidence that this can be achieved through improving the accuracy of blood loss volume measurements. The diagnosis may rely on factors other than volume, such as speed of blood flow and nature of loss. A change in direction of future research is required to explore these in more detail.

**Electronic supplementary material:**

The online version of this article (doi:10.1186/s12884-015-0653-6) contains supplementary material, which is available to authorized users.

## Background

Postpartum haemorrhage (PPH) is the single most common cause of maternal mortality globally, with 99 % of all maternal deaths occurring in low-income countries [1]. In many high-income countries the incidence of severe PPH is increasing and is the leading cause of severe maternal morbidity [2–4]. Primary PPH is defined as blood loss of 500 ml or more [5], and severe PPH as 1000 ml or more [6], within 24 h of childbirth. PPH is most frequently attributed to uterine atony [6], which usually occurs within the first hour of birth and can escalate rapidly [7].

Following an earlier systematic review of causes of maternal death, Khan et al. (2006) concluded that most deaths from PPH could be avoided by prevention strategies, appropriate diagnosis and management [8]. However, while World Health Organization (WHO) Guidelines [5] include thirty three recommendations for the prevention and management of PPH, there is only one recommendation for diagnosis. This is for regular assessment of uterine tone in the two hours immediately following birth to promote early detection of uterine atony.

Delays in the diagnosis and treatment of PPH are believed to have a direct effect on the severity of bleeding, the development of complications such as coagulopathy and resulting rates of morbidity and mortality [9, 10]. Delays are reported to be caused by misinterpretation of the extent of blood loss and its physiological effects, failure to recognise hidden bleeding, and failure to escalate care to more senior colleagues [9]. Experts [11] have suggested that improving the accuracy and reliability of blood loss estimation is the ‘crucial step’ in early detection of PPH.

Visual estimation of blood loss has been described as the most common and practical way to quantify maternal blood loss, particularly following vaginal birth [12]. However, the method is generally accepted to be inaccurate [13] and there appears to be little consensus on how this situation can be improved.

An integrative literature review was conducted to explore postnatal blood loss assessment and to assess the success of strategies used to evaluate blood loss following childbirth. The review was conducted and reported in line with the standards of the PRISMA statement (Preferred Reporting Items for Systematic Reviews and Meta-Analyses) [14]. A systematic review of strategies to evaluate maternal blood loss during childbirth' or Registration number CRD42013004738

## Methods

### Integrative review methodology

A 5-stage process for conducting integrative reviews [15], developed from an established method for conducting systematic reviews [16], was used to guide this review and comprised the following five stages: problem identification, literature review, data evaluation, data analysis (including data reduction, data display, data comparison, conclusion-drawing and verification), and presentation.

### Problem identification

The focus of the integrative review was to explore strategies and methods of blood loss assessment used at childbirth, and to determine their usefulness in improving the accuracy and reliability of blood loss estimation and preventing delays in PPH diagnosis.

### Literature review strategy

An electronic search was conducted between April and June 2013, with no restrictions applied. Databases included Embase (1974–2013), Medline and CINAHL Plus (1937–2013), the British Nursing Index (1997–2013), Maternity and Infant Care (1971–2013), and PsycINFO (1806–2013). The terms: ‘blood loss’, ‘postpartum haemorrhage’ and ‘postpartum bleeding’ were combined separately with the keywords: estimation, measurement, quantification, assessment, accuracy, prevention, diagnosis, gravimetric method, spectrophotometry, alkaline haematin method, training, guideline, protocol, technology, scenario, simulation, recognition, decision-making, practice, competence and blood collection bag. An additional internet search identified evidence-based policy documents on recognition and management of PPH. The reference lists of all retrieved papers were checked and identified an additional 20 papers.

### Inclusion and exclusion criteria

**Fig. 1:**
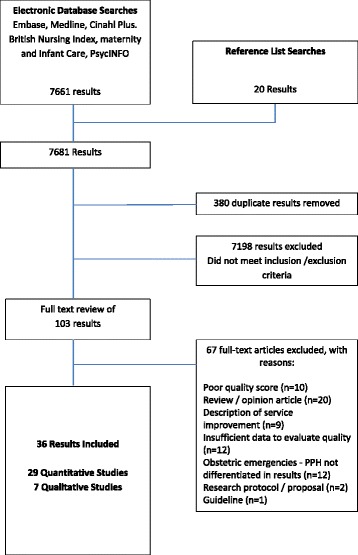
Screening and Inclusion Process

A total of 7681 papers were identified by the search methods. One reviewer (AH) screened all titles and abstracts against the inclusion/exclusion criteria. Where no abstract was available the full paper was retrieved and screened. Studies were excluded if they focussed on secondary PPH; definition of risk factors for PPH; and treatment regimens for PPH. Following removal of duplicates and papers not meeting the inclusion criteria, 103 papers remained. These 103 papers were read in full, independently appraised and quality scored by two reviewers (AH & TL or AH and ADW). The screening and inclusion/exclusion process is summarised in Fig. 1.

### Data evaluation

Appraisal and quality scoring were documented using published tools appropriate to the study design [17–20]. Each quality appraisal tool was selected, adapted and piloted by the reviewers to ensure consistency in assessment and grading of studies between reviewers Quality assessment of the quantitative studies was difficult as they were mainly observational studies and there was no definitive tool that would suit them all. Therefore existing tools were adapted and piloted and used to provide a systematic approach to evaluating each study before discussion between the reviewers. Authors’ of two studies were contacted to clarify aspects of their study design to determine eligibility for inclusion. The decision to include studies in the review was based on the consensus of opinion of two reviewers, with the opinion of the third reviewer sought if there was any disagreement.

Consistent appraisal of the qualitative data was achieved through the use of comprehensive criteria produced by Walsh and Downe [17], which were piloted and used as a checklist. A quality score was assigned to studies using the scoring system adapted by Downe [19] (available upon request from authors). From 19 studies considered for inclusion, 6 were excluded as they did not meet the inclusion criteria. Thirteen were critically appraised and scored independently by two reviewers. Disagreements were resolved through discussion, resulting in the upgrading and inclusion of one study [49]. Six studies with a quality rating of ‘D’ were rejected outright. The characteristics of the remaining 7 included studies scoring A-C are shown in additional file 1. The suitability of the papers for metasynthesis was considered but because of the overall low quality of the studies deemed to be inappropriate and they were presented as a narrative review instead.

### Data analysis

The papers were manually organised into subgroups based on interventions and research design. A narrative review of each study was recorded in Microsoft Word before being summarised in a table format. The tabulated summary facilitated comparisons between the studies and enabled preliminary conclusions to be drawn. Papers were then re-examined by the authors in relation to these conclusions to further explore as well as verify the findings. Interpretation of findings were discussed and agreed between all members of the review team and a narrative synthesis was produced in relation to key findings.

## Results

Thirty-six papers were included in the integrative review. They used a range of methodological approaches that are summarised in Table 1. An additional file shows the characteristics of included studies in more detail (See Additional file 2). Twenty-nine quantitative studies are included that tested a range of blood loss evaluation methods (See Table 2). Twenty–four tested the diagnostic accuracy of visual estimation with 16 of these testing strategies aimed at improving the accuracy of estimation. A further 5 studies evaluated a combination of interventions aimed at reducing the incidence of PPH and improving its management. Four studies used a randomised controlled design. Seven qualitative studies were included (See Table 3). Four of these were conducted in low-income settings and explored community perceptions of blood loss. Of the 3 qualitative studies conducted in high-income settings, 2 explored women’s experiences of PPH and PPH-related hysterectomy, and 1 explored midwives’ management of the third stage of labour.

**Table 1:** Types of research methodology in the 36 included papers

Type of Research	Number of Studies	Name of Lead Researcher
Observational Studies	22	Al Kadri, Audureau, Bose, Brant, Brant, Buckland, Dildy, Duthie, Duthie, Gharoro, Glover, Kavle, Larsson, Maslovitz, Newton, Razvi, Stafford, Tixier, Toledo, Toledo, Wangwe, Yoong
Randomised controlled trial	4	Denaux-Tharaux, Patel, Toledo, Zhang,
Audit	3	Dupont, Dupont, Rizvi
Qualitative – no specified perspective	4	Jangsten, Kalim, Vaate, Sibley
Naturalistic Inquiry	1	Elmir
Husserlian phenomenology	1	Mapp
Grounded theory	1	Matsuyama

**Table 2:** Blood loss evaluation methods tested in the 29 quantitative studies

Methods	Number of Studies	Name of Lead Researcher
Visual estimation	9	Bose, Buckland, Dildy, Glover, Maslovitz, Toledo, Toledo, Toledo, Yoong
Visual estimation and spectrophotometry	7	Brant, Brant, Duthie, Duthie, Larsson, Newton, Razvi
Weighed blood loss and spectrophotometry	1	Kavle
Visual estimation and gravimetric (weighed)	1	Al Kadri
Visual estimation, blood collection bag and spectrophotometry	1	Patel
Visual estimation and maternal haematocrit	3	Gharoro, Stafford, Wangwe
Blood collection bag and maternal haematocrit	1	Tixier
Clinical diagnosis and haemoglobin	1	Dupont
Clinical diagnosis and blood collection bags	1	Audureau
Visual estimation and blood collection bag	1	Zhang
Mulitfaceted – audit, protocol and training	1	Denaux-Tharaux
Audit and training	2	Dupont, Rizvi

**Table 3:** Phenomena of interest in the 7 qualitative studies

Phenomena of Interest	Number of studies	Name of Lead Researcher
Knowledge, attitudes and practices of birth attendants and community members related to bleeding, PPH and care-seeking behaviour	4	Vaate, Sibley, Matsuyama, Kalim
Women’s experiences of PPH	2	Mapp, Elmir
Midwives experiences and rationale for third stage management	1	Jangsten

In 1959 Wilcox et al. [21] used the cyanmethemoglobin photelometric method of measuring blood loss and determined that average blood loss in 25 women undergoing caesarean section was 1028 ml. He noted that in 22 cases the clinical estimate of blood loss was 325 ml less than the laboratory measured values and was overestimated in just 3 cases. The method was described as a ‘time consuming, exacting procedure’ (p535), and although deemed unsuitable for clinical use, was commended for research purposes. Spectrophotometry was subsequently developed as the ‘gold standard’, laboratory-based technique for calculating blood loss. With an error rate of between zero and 10 % it was considered to be a reliable method for quantifying blood loss in the research context [22].

Eight studies [12, 23–29] used spectrophotometry to determine average blood loss at vaginal and caesarean birth. Mean blood losses at uncomplicated vaginal births were reported as 226 ml [28] to 286 ml [12, 29]. Two studies of women, with ‘additional complications’, having elective and emergency caesarean section reported mean blood losses of 1068 ml and 1,106 ml respectively [24, 25]. Later studies of women undergoing elective caesarean section reported a mean blood loss of 487 ml [27] and a median loss of 500 ml [12].

Seven [12, 23–28] of the 8 studies found that visual estimation was inaccurate. One study [23] reported that there was no correlation between estimated and measured loss at vaginal delivery, while 4 studies reported underestimations of between 46 % and 75 % [24–28]. Six out of eight studies confirmed that the extent of underestimation increased as the volume of blood loss increased [23–28]. Two studies [25, 28] reported that estimation was most accurate at low volumes, although there was also a tendency to overestimate small amounts. Only one study [29] found ‘visual estimates’ to be accurate (to within 4.90 ml of the spectrophotometry values). However, the paper described that blood loss was collected 'on a delivery pad… and was then weighed on a scale” (p25), suggesting that the gravimetric method was compared to spectrophotometry, and not visual estimation.

The gravimetric method was used to assess the accuracy of visual estimation in 150 vaginal births and found that visually estimated blood losses were 30 % lower than the gravimetric estimates across all professional groups, irrespective of their levels of experience [30]. Three studies compared changes in maternal haematocrit values to visually estimated blood loss [31–33]. The first study [31] used changes in haematocrit values within a formula for calculating estimated blood loss and found statistically significant underestimation of blood loss in 677 women when using visual estimation. While the second study [32] found that all women with estimated blood loss greater than 500 ml had a decline in their haematocrit at 48 h post-delivery, a third study [33] found that there was a lower incidence of PPH (PPH rate: 8.9 %) when using visual estimation compared to using a fall in maternal haematocrit values (PPH rate: 16.2 %).

Nine studies aimed to improve skills and accuracy in visual estimation through training, education and development of clinical assessment tools [34–42], although none were able to show long term improvements or translation of skills into clinical practice. One study [36] photographed each reconstruction of blood loss to produce a pictorial guide for blood loss assessment although this was not evaluated in clinical practice.

One study evaluating estimations of eight blood loss simulations before and after a PowerPoint presentation on blood loss estimation found that error was reduced in the post-presentation estimates [35]. However, a similar study that found improved blood loss estimates immediately after didactic and web-based training [40] retested the web-based training group after 9 months and found that accuracy of blood loss estimation had deteriorated [42].

A study using high fidelity PPH simulation [39] found that midwives and obstetricians visually underestimated blood loss by 40 % to 49 %. In an intervention aimed at improving the accuracy of estimation, a small number of teams were given verbal instruction to estimate blood loss at set intervals. The accuracy of estimation was found to improve in the intervention groups although blood losses were still underestimated by an average of 32 %. The simulations were videotaped and, at the conclusion of each scenario, played back to participants who discussed their actions. A ‘verbal questionnaire’ was used during the discussion to ask specific questions about PPH management, the answers to which were quantitatively analysed. It was reported that 68 % of participants said they deliberately increased their estimations because they knew that visual estimation underestimated blood loss. A number of participants said that they would consider other parameters when making decisions during PPH such as the haemodynamic stability (34 %) of the woman, laboratory results (42 %) and pad counts (26 %). Many participants described that they used ‘gut feeling’ (60 %) or ‘pure guesswork’ (32 %) to estimate blood loss. Although these comments were not explored any further by the authors of the study, who dismissed them as *‘*disturbing*’* (p933), they may suggest that factors other than volume are important during decision-making about blood loss.

A small number of qualitative studies suggested that factors other than volume were taken into consideration when making decisions about blood loss. Four studies [43–46] in low income countries included trained and untrained traditional birth attendants, women, their families and community members and one study in a high income country included Swedish midwives [47], described blood loss at birth as being normal to some extent.

“I was taught a little bleeding is normal at this stage” [47] (p613).

While in one study [43] food cans were described as being used to collect and quantify blood loss, all 4 studies in low income settings described ‘abnormal’ or ‘alarming’ blood loss using language that depicted the speed and flow of the blood, rather than the volume:

“A continuous, swift flow or gush… it overflows the place…it comes out with force, gushing like water comes from a tube well” [44] (p355)

Delays in the diagnosis of PPH were also apparent in the low income settings where maternal collapse, loss of consciousness, pallor and cyanosis were used as signs that blood loss was serious [45]. The findings of two studies [48, 49] involving women in the high income settings also highlighted the clear perceptions women have at the time of haemorrhage.

Four studies evaluated guideline compliance and the impact of educational interventions on clinical outcomes [50–53]. Two studies reported only partial compliance with local and regional guidelines which was improved by one author [50] through a programme of focussed training for all staff providing intra-partum care alongside revised guidelines. Similarly, a ‘persistent reduction in the prevalence of severe PPH’ (p583) was reported by an observational study [54] in France, where the management and quality of care in all cases of severe PPH was subject to regular audit. However, the authors stated that it was unclear whether it was regular audit or other unrelated factors (such as global improvements in PPH management or a reduction in the number of women at risk) that had led to the decreased incidence of severe PPH. The remaining two studies [52, 53] used a multifaceted approach to guideline implementation and training with the aim of improving PPH prevention, recognition and management. There were some changes in clinical practices as a result of the interventions. In the first study [52] there was improvement in use of second line uterotonics and escalation of care. In the second study [53] active management of the third stage of labour increased from 58.8 % to 75.9 %, and systematic use of blood collection bags increased from 3.9 % to 76.4 %. However, the incidence of major PPH and initial responses to it were reported to be unchanged and the authors concluded that the impact of blood collection bags on clinical outcomes could not be demonstrated.

Four studies [13, 38, 55, 56] specifically investigated the effect of blood collection bags on the accuracy of blood loss estimation, PPH diagnosis, and PPH management. Calibrated bags were shown to facilitate accurate estimations in both clinical reconstructions [38] and in clinical practice [13]. Patel et al. (2006) [13] compared visual estimates with calibrated blood bag estimates before testing a sub-group of 10 blood collection bags using spectrophotometry. Visual estimates were 33 % lower than the blood bag estimates, 10 of which were verified using spectrophotometry. However, while Patel et al. (2006) showed that blood collection bags provided an accurate assessment of blood loss, a later study [56] of 122 women found that, for blood losses of 500 ml or more, the positive predictive value of the blood collection bag was only 66.7 %. The authors suggested that lowering the diagnostic threshold of PPH to 300 ml may be necessary to improve the diagnostic value of the collection bag. A cluster randomised trial [55] including 25,381 vaginal deliveries in 13 European countries is the largest study to date comparing the use of the blood collection bag to visual estimation. The study proposed that using blood collection bags would facilitate more objective monitoring and measurement of postpartum blood loss than visual assessment, triggering an earlier response from caregivers and thus reducing the incidence of severe PPH. Severe PPH occurred in 189 of the 11,037 vaginal births using blood collection bags in the intervention group (1.71 %); and 295 of the 14,344 vaginal births using visual assessment in the control group (2.06 %). At both individual and cluster level analysis, the difference was not statistically significant and the authors concluded ‘that the systematic use of a collector bag after vaginal delivery did not modify the rate of severe postpartum haemorrhage’. The authors’ offered a number of explanations for what they describe as the ‘negative result’ of their trial, summarised by the statement that,

“… having a more accurate assessment of postpartum blood loss is not by itself sufficient to change behaviours of care givers and improve the management of PPH” (p.6).

## Discussion

Experts [11] have proposed that improving the accuracy and reliability of blood loss estimation is a ‘crucial’ step in early detection of PPH; and that most deaths from PPH could be avoided through ‘appropriate diagnosis’ [8]. This review has examined strategies currently used for quantifying blood loss and considered their usefulness in achieving these aims. The review has shown that while a small number of strategies did achieve more accurate and reliable estimations of blood loss in practice, none had any significant impact on the timing of PPH diagnosis or prevented progression of blood loss to severe PPH.

An integrative literature review was considered the most appropriate method for this review as the method allows for inclusion of studies using a range of methodological approaches [57, 58]. The synthesis of such a diverse range of studies has the potential to develop new knowledge and perspectives on topics that relate to clinical practice, and can help identify areas for future research [57]. The integrative review process was conducted according to a clear methodology [15] and guided by a pre-defined protocol. A systematic approach was taken by a team of reviewers to search, select, critique and interpret the range of studies exploring blood loss evaluation. However, there are a number of limitations which should be considered. Combining diverse methodologies can be a complex process which may contribute to bias and inaccuracies in the conclusions that are drawn from such reviews [59]. This issue may be exacerbated by the variable quality of the studies included. Many studies used small sample sizes or samples that were described as ‘convenience’, ‘opportunistic’ and ‘voluntary’. In addition, many of the study designs reduced the reliability and generalisability of the results. Despite this, many authors made claims and recommendations that went beyond their findings and the scope of their studies. All these factors required careful consideration when interpreting the studies in the review. However, such limitations were considered carefully and balanced against the advantages of including the range of studies that have contributed to the construction of knowledge in this area and informed the direction of research over the last 50 years. A comprehensive search of online databases of systematic reviews revealed that no other reviews were in progress or had been previously completed on this topic. One previously published literature review [59] had concluded that a combination of direct measurement and gravimetric measures were the most practical and reliable to use to provide an accurate measurement of blood loss. However, questions remained unanswered about whether such methods were useful in the early detection of PPH.

The small study conducted by Wilcox et al. in 1959 [21] was influential, not only for introducing a scientific method for quantifying blood loss at birth, but also for shaping knowledge and attitudes to blood loss evaluation and influencing the direction of research that followed. The study not only showed that average blood loss at birth could be reliably established but, more fundamental to this review, that clinical estimates of the volume of blood loss were unreliable. The series of diagnostic accuracy studies that followed reiterated and built on these findings. The rationale for Brant’s (1966) [24] replication of such a study was captured by his citation of Wright (1961) who, Brant stated, had found:

*“…that failure to recognize the seriousness of haemorrhage at Caesarean section was at times a major factor in maternal death” (p456).*

The sentiment of this statement was reiterated by many study authors and indeed by recent experts. However, this review suggests that the apparent link between estimation of blood loss as a volume, and delays in recognition and treatment of PPH is a tenuous one and raises a series of questions.

Patel et al. (2006) [13] described visual estimation of blood loss as the:

“current worldwide standard practice of postpartum blood loss assessment (where) a minimally trained health care provider generally observes blood lost during delivery and makes a quantitative or semi-quantitative estimate” (p221).

What was never questioned by Patel (2006) or other researchers however, is when or whether this quantitative estimate is used specifically for the purposes of PPH diagnosis. The findings of this review would suggest that it is not. In the qualitative studies, practitioners used the speed and nature of flow more than the estimated volume to determine the severity of the PPH. Furthermore, the majority of studies that tested the accuracy of visual estimation of blood loss found that practitioners appeared to normalize blood loss. Whilst small volumes tended to be overestimated, the method generally underestimated the volume of blood loss with the size of discrepancies increasing with increasing levels of blood loss. Inaccuracy was common across all professional groups, with levels of experience of blood loss estimation making no difference to skills in the method. Additional training did not improve skills of estimation in the long term and were not shown to have an effect on clinical outcomes. Authors of just two studies suggested that a PPH diagnosis could be missed or was not detected by visual estimation of blood loss [28, 33]. Razvi et al. (1996) [28] suggested that the reason was that ‘estimates simply reflected teaching about what constitutes average blood loss at birth’(p154).

Current theory about PPH diagnosis appears to suggest that the diagnosis of PPH occurs as a linear process, preceded by quantification of blood loss as a volume and followed by actions determined by the extent of the loss. The focus of the majority of studies in this review has therefore been based on the premise that better estimation will lead to earlier recognition, and earlier recognition will lead to improved outcomes. This theory may have been refuted by the large RCT that evaluated the use of blood collection bags. While the accuracy of quantifying blood loss volume in real time was facilitated through the use of the collection bags, their use did not result in a reduced incidence of severe PPH. Clinical decision–making and the factors affecting the progression of PPH are likely to be complex and the notion that the situation could be improved by more accurate visual assessment of blood loss may be unrealistic. The ability to recognise high-risk situations (e.g. underlying medical disorders, emergency second stage CS, or placental disorders), and having an efficient care pathway in place to deal with any resulting PPH, may be far more important for improving outcomes.

No studies using mixed methods were identified by this review. It appears to have been a disadvantage that none of the studies presented contained a qualitative element that explored the use of the strategies studied. Instead, study authors generally presented their own plausible explanations for their results and hypothesised why their interventions did not work, without the benefit of qualitative data. Given that quantitative research generally only provides numerical data, it is often very helpful to have qualitative data from the same study to aid in its interpretation. If there is only quantitative data then the interpretation is purely based on the authors’ own assessment, with all their inherent biases. Often the authors get it right, but in this situation academics have considered that PPH diagnosis and treatment is linear and starts with volume assessment in order to make an initial diagnosis of PPH. In contrast, the qualitative data (and the experience of many clinicians) is that the numerical quantification of blood loss volume is usually retrospective and plays only a small part in on-going management. The initial diagnosis is usually made on clinical impression alone, with the rate of blood flow and the woman’s physiological response to the loss used to govern on-going management. Several indices have been developed to quantify the physiological response including the Shock Index (pulse/systolic blood pressure), which has recently been shown to closely correlate with adverse maternal outcomes [60, 61].

From the women’s perspective, many were acutely aware of the extent of their own blood loss and highly perceptive of the non-verbal communication of those around them. Their contribution to the decision-making process should not be overlooked. Future research should focus on addressing the gaps identified in this review. Future definitions of PPH may need to focus less on absolute volume of blood loss, and more on physiological signs, as highlighted by the recent work on shock index [60, 61]. Research should now focus on developing our understanding of blood loss evaluation from the perspective of those involved in the process. This will act as a catalyst to identifying and developing new and innovative strategies to support decision-making in clinical practice.

## Conclusions

This integrative review has revealed that most studies attempting to improve recognition and response to PPH have focussed on improving volume estimates of blood loss. However, it should not be assumed that because PPH is defined as a volume, PPH diagnosis occurs as a response to volume. The lack of qualitative research in this area means that factors that affect decision-making during PPH diagnosis and the usefulness of the methods of measurement presented have not been explored. The small amount of qualitative evidence available suggests that the nature and speed of the blood loss as well as the condition of the woman are important factors in the decision-making process. It is likely that recognition of a clinically important postpartum haemorrhage at childbirth is a complex, dynamic, process and that estimation of blood loss volume plays only a small part.

## Additional files

Additional file 1:
**Characteristics of all included studies.** (PDF 246 kb) Additional file 2:
**Characteristics of qualitative studies included after quality review.** (PDF 241 kb)

## Abbreviations

PPH: Postpartum hemorrhage
WHO: World health organization
