# Supplementary material for: Is accurate and reliable blood loss estimation the 'crucial step' in early detection of postpartum haemorrhage: an integrative review of the literature
Source: BMC Pregnancy Childbirth. 2015 Sep 28;15:230. doi: 10.1186/s12884-015-0653-6 (PMC4587838; doi:10.1186/s12884-015-0653-6)
Supplement: Additional file 1: — Characteristics of all included studies. (PDF 246 kb) [file 12884_2015_653_MOESM1_ESM.pdf]

Additional file 2: Characteristics of all included studies

Lead author/year/country	Sample	Study purpose	Study Design	Methods of blood loss evaluation	Summary of findings relevant to review
**Accuracy of visual estimation in clinical setting**					
Newton 1961 USA	100 women without complications, vaginal birth, receiving oxytocin or no oxytocin at birth	To compare effect of oxytocin on blood loss and estimated and measured loss.	Observational Study	Visual estimation and spectrophotometry	Small blood losses were overestimated, large blood losses were underestimated
Brant 1966 UK	36 women, intrapartum (n=14) or elective (n=22) caesarean section	To determine blood loss during caesarean section. Comparison of estimated and measured loss.	Observational Study	Visual estimation and spectrophotometry	Mean measured blood loss at caesarean section 1106ml (emergency), 1068ml (elective). Estimates approx. half the measured amount.
Brant 1967 UK	57 women, spontaneous and assisted vaginal birth with estimated blood loss greater than 200ml.	To compare estimated and measured loss	Observational Study	Visual estimation and spectrophotometry	Underestimation increased when recorded loss above 300ml. Serious discrepancies with large losses.
Duthie 1990 Hong Kong	62 women, uncomplicated pregnancies, spontaneous labour, vaginal birth	To compare estimated and measured blood loss	Observational Study	Visual estimation and spectrophotometry	Mean measured blood loss: Primips 401 ± 29ml; Multips 319 ± 41ml. Mean estimated loss significantly lower than mean measured loss. Underestimation increased as blood loss increased.
Duthie 1992 Hong Kong	40 women, singleton pregnancies, elective caesarean section under GA with transverse or midline incisions	To compare estimated and measured intra-operative blood loss at caesarean section.	Observational Study	Visual estimation and spectrophotometry	Mean measured blood loss 487ml. Mean estimated blood loss 425ml. Overestimation of blood losses <400ml. Underestimation increased when blood loss >600ml.
Razvi 1996 Singapore	111 women, singleton pregnancies, spontaneous labour, vaginal birth	To compare estimated and measured blood loss	Observational Study	Visual estimation and spectrophotometry	Mean measured blood loss 226.7ml. Mean estimated blood loss 255.3ml. Overestimation of blood losses <150ml by >20%. Blood loss 301-500ml underestimated by >20%
Kavle 2006 Tanzania	155 women (including 3	To accurately measure	Observational Study	Weighed blood loss	Median blood loss at birth

	twin pregnancies), vaginal birth, hospital facility	blood loss at birth in a low income country.		(described as ‘estimates’) and spectrophotometry	153ml; birth to 24 hours 112ml; total 286ml. Mean difference 4.90ml. Imprecision greater when blood loss >200ml
Larrson 2006 Sweden	55 women, 29 planned and unplanned caesarean sections, 26 vaginal births	To validate estimation of blood loss at delivery	Observational Study	Visual estimation and spectrophotometry	Median measured blood loss:<br>Vaginal birth 254ml<br>Caesarean 440ml<br>Median estimated blood loss:<br>Vaginal birth 325ml<br>Caesarean 500ml<br>No correlation between pre and post birth haemoglobin values and measured or estimated blood loss.
Patel 2006 India	123 women, vaginal birth, district hospital	To compare visual estimation to blood collection drape estimation and a subgroup of drape estimations to spectrophotometry	Pilot Randomised Controlled Trial	Visual estimation, blood collection drape and spectrophotometry	Mean blood losses:<br>Visual estimation 203ml<br>Drape estimation 304ml<br>Mean difference between drape and spectrophotometry 51ml.<br>Correlation for two methods was high
Stafford 2008 USA	677 women, all types of birth.	To compare pre- and post-birth haematocrit; visually estimated blood loss and calculated blood loss.	Observational Study	Visual estimation<br>Pre and post birth haematocrit values<br>Calculated blood loss	Visual estimation statistically different from calculated blood loss in all births. Underestimation increased as blood loss increased.
Gharoro 2009 Nigeria	153 women, vaginal birth	To compare visually estimated blood loss to changes in intrapartum and post birth maternal haematocrit values	Observational Study	Visual estimation and maternal haematocrit values	Mean blood losses:<br>Visual estimation 217ml (PPH rate 8.9%)<br>Mean postpartum haematocrit change -0.74 to ±3.99 (-12 to -4 when blood loss ≥ and -10 to +8 in blood loss <500ml)
Zhang 2010 Europe	78 maternity units (25,381 women, vaginal	To evaluate effectiveness of systematic use of a	Cluster Randomised Controlled Trial	Visual estimation, blood collection bag	Severe PPH occurred in 189 of 11,037 vaginal

	birth)	transparent blood collection bag to measure postpartum blood loss after vaginal delivery in reducing the incidence of severe PPH.			deliveries (1.71%) in intervention group and 295 of 14,344 in control group (2.06%). Not statistically significant at individual or cluster level analyses. Compared with visual estimation the use of blood collection bag did not reduce rate of severe PPH.
Tixier 2011 France	122 women, singleton pregnancy, >37 weeks, cephalic presentation.	To evaluate the validity of using an under-buttock blood collection bag for PPH diagnosis	Observational Study	Blood collection bag, maternal haemoglobin and haematocrit changes.	Mean blood loss: 233.15ml. At 500ml, sensitivity of the collection bag was 6.7%, specificity was 94.2%. Positive predictive value (PPV) was 66.7% and negative predictive value 94.2%. At 300ml sensitivity was 88.9%, specificity was 82.7%, the PPV was 47% and the NPV was 97%.
Al Kadri 2011 Saudi Arabia	150 women, vaginal birth	To compare estimated and measured blood loss	Observational Study	Visual estimation and gravimetric method	Significant differences between measured and estimated blood loss. Visual estimates underestimated blood loss by 30%
Wangwe 2012 Tanzania	395 women, vaginal birth between 0800 and 1500hours	To compare visually estimated blood loss to changes in pre and post birth maternal haematocrit values	Observational Study	Visual estimation and maternal haematocrit values	Mean blood loss:<br>Visual estimation 164.9ml (PPH rate 8.9%)<br>Mean postpartum haematocrit change 5.3 (-0.48 to 19.7, PPH rate 16.2%)
**Clinical reconstructions and simulation of blood loss**					
Glover 2003 Australia	26 maternity health care professionals	Visual estimation of 6 simulated clinical blood loss scenarios	Observational Study	Visual estimation	Medical staff (n=5) most accurate. Midwives (21) accurate up to 300ml. Overall, volumes

					underestimated by 30-50%.
Dildy 2004 USA	52 medical students and doctors; 1 obstetric nurse	Visual estimation of 7 simulated clinical blood loss scenarios pre and post training	Observational Study	Visual estimation	Extent of overestimation of small volumes and underestimation of large volumes was reduced after training. Years of clinical experience did not influence accuracy.
Bose 2006 UK	103 maternity health care professionals	Visual estimation of 12 simulated clinical blood loss scenarios	Observational Study	Visual estimation	Significant underestimation of blood loss occurred in 5 out of 12 scenarios. None were significantly overestimated.
Toledo 2007 USA	106 maternity health care professionals	Visual estimation of blood loss using calibrated and un-calibrated under-buttock blood collection bags	Crossover Randomised Controlled Trial	Visual estimation	No difference in gender, level of training or experience. Underestimation proportional to volume and greater when viewing non-calibrated bags first. Inaccuracy increased with increasing blood volume.
Buckland 2007 Australia	88 maternity health care professionals	Visual estimation of 5 simulated clinical blood loss scenarios	Observational Study	Visual estimation	Estimation most accurate at lower volumes (350ml) and when calibrated container used. Accuracy decreased once blood soaked into clothing, linen or sanitary pads.
Maslovitz 2008 Israel	68 doctors and 80 midwives divided into 50 teams	Visual estimation of blood loss. Visual estimation at 3 set intervals in 8 intervention teams	Observational Study	Visual estimation	Underestimation of blood loss by 40%-49% in standard teams and 32% in intervention teams. Experience did not influence accuracy.
Yoong 2010 UK	47 maternity health care professionals	Visual estimation of small volumes of blood loss on 5 sanitary pads	Observational Study	Visual estimation	All 4 groups showed an overall tendency to overestimate. No statistically significant differences among

					professional groups.
Toledo 2010 USA	372 maternity health care professionals. Live training 231; web training 141	Visual estimation of 5 simulated clinical blood loss scenarios pre and post training	Observational Study	Visual estimation	Improvement in the immediate post-test accuracy of blood loss estimations ($p<0.001$). Small volumes overestimated, large volumes underestimated. Post-test estimates were more accurate for volumes that most closely approximated those used during the pre-test training sessions.
Toledo 2012 USA	52 participants recruited from 141 web-training participants in Toledo (2010) study	To assess skill decay in visual estimation of blood loss 9 months after initial web-based training	Observational Study	Visual estimation	The median (IQR) aggregate pre-test accuracy of blood loss estimation was -47.8% (-60.9 to 28.7%) with an improvement to -13.5% (-18 to -9.8%) in the immediate post-test ($p<0.001$). At the 9 month follow-up, accuracy had decreased to -34.6% (-53.4% to -14.3%).
**Multifaceted interventions to improve PPH recognition and management**					
Dupont 2009 France	16 maternity units (1144 cases of PPH occurring out of 21,350 births)	To evaluate regional guideline compliance during management of PPH.	Audit	Clinical diagnosis<br>Maternal Haemoglobin	Clinical diagnosis in 82.5% of severe cases and 77.5% of non-severe cases. 1 out of 5 cases diagnosed by lab tests during 1st 3 postnatal days. Regional guidelines only partially followed.
Audureau 2009 France	19 Maternity Units	To evaluate effectiveness of a multifaceted intervention on practices for prevention, diagnosis and management of PPH and on the prevalence of major PPH	Observational Study	Clinical diagnosis<br>Blood collection bags	Increase in active management of third stage of labour (from 58.8% to 75.9%) and use of blood collecting bags (3.9% to 76.3%). Reduction in delay in 2nd line drug treatment and instrumental

					examination. Increase in haemoglobin measurements after PPH; use of surgery; and blood transfusions. No change in prevalence of major PPH and initial PPH management.
Denaux-Tharaux 2010 France	106 maternity units (146, 781 births)	To test the hypothesis that a multifaceted intervention aimed at increasing the translation into practice of a protocol for early management of PPH, would reduce the incidence of severe PPH.	Cluster-randomised Controlled Trial	Intervention comprised outreach visits to discuss protocol in local context, reminders, peer reviews of severe PPH incidents	No significant difference in mean rate of severe PPH. Some elements of PPH management were more frequent in intervention units. The educational intervention did not affect the rate of severe PPH but improved some practices.
Dupont 2011 France	2 maternity units	To assess the impact of regular criteria-based audits on the prevalence of severe PPH and quality of care	Audit		Significant reduction in prevalence of severe PPH. Improved quality of care. Proportion of cases with optimal management increased. Deviations from recommended care: delayed or no examination of the uterine cavity, absence of instrumental exam of vagina and cervix, and delayed or no administration of second line uterotonics.
Rizvi 2004 Dublin	54 cases of massive PPH before intervention and 15 cases following.	To evaluate management of PPH before and after guideline revision dissemination and introduction of drill training	Audit	Not stated	100% guideline compliance in all 15 cases of massive PPH following intervention.
**Experiences of blood loss and blood loss estimation**					
Vaate 2002 The Gambia	22 trained traditional birth attendants (TBAs) and their 2 supervisors in 12 villages	To assess knowledge, attitudes, practices, and role of TBA's in prevention, recognition and	Semi-structured interviews and group discussions	Not applicable	TBAs indicated that they observed blood loss carefully following delivery. 'Normal'

		management of postpartum haemorrhage			bleeding was restricted to the area of birth. ‘Alarming’ blood loss was described as “bleeding with force”; “pulsating”; “bleeding like an open tap” and “bleeding all over the place”.
Mapp 2005 UK	10 women who responded to radio and newspaper advertisement	Women’s ‘lived experience’ of obstetric emergencies	Face to face 1:1 Interviews	Not applicable	Theme of communication highlights that women interpret the non-verbal cues of health professionals. They described feeling reassured by a smile or having fears perpetuated by the facial expressions of the staff.
Sibley 2007 Bangladesh	20 participants from 4 groups: women of reproductive age, women between 50 and 70 years living in the extended family; trained and skilled birth attendants.	To identify local cultural theories that women and (untrained) TBAs use to recognise and care for postpartum problems, including PPH.	Face-to-face semi-structured interviews and free-listing.	Not applicable	PPH not considered a common problem but participants found it difficult to define. Bleeding was quantified using kilograms, sheer (a local measure of weight), litres and counts of blood soaked objects e.g. pads, clothes, buckets, jute mats and saris.
Matsuyama 2008 Nepal	28 participants from following groups: village wise men, wise women, village health volunteers, mothers-in-law, women with small children, husbands, TBAs, traditional healers and pharmacists.	To explore local understanding of bleeding during pregnancy and at delivery, and the meaning attached to it.	Semi-structured interviews, free-listing, ranking and pile sorts	Not applicable	Bleeding after birth viewed as normal and even beneficial. Even when considered excessive, care-seeking is often delayed while natural remedies are used.
Kalim 2009 Bangladesh	168 participants (women of reproductive age (15-49 years) and elderly women (50-70 years)).	To explore the differences in perceived knowledge and care-seeking behaviours of women in	Free listing, rating exercises, hypothetical case scenarios and in-depth interviews	Not applicable	Bleeding at birth considered normal and good for the woman as blood associated with

		relation to PPH or eclampsia			'pollution'. Retained placenta recognised as life threatening with severity being related to blood loss. Signs used to recognise the seriousness of blood loss included collapse and unconsciousness.
Jangsten 2010 Sweden	32 midwives	To explore midwives' experiences of management of the third stage of labour and assessment of risk.	Focus groups	Not applicable	Management of the 3rd stage of labour varied. Not all midwives agreed with prophylactic oxytocin in the 3rd stage, particularly following normal birth. Decisions were based previous experience, hospital guidelines, risk assessment and sensitivity to women's needs.
Elmir 2012 2012	21 women self-selected following a media release, posters and flyers in a range of public locations.	To describe women's experiences of having an emergency hysterectomy following a severe PPH	Face-to-face, telephone and internet email interviews	Not applicable	Women felt 'despair' at the amount of blood loss and recalled vivid memories of events. Many experienced flashbacks and nightmares. Despite the fact that many women 'felt close to death', they were still able to perceive the anguish of those around them, particularly their relatives.
