# Supplementary material for: Is accurate and reliable blood loss estimation the 'crucial step' in early detection of postpartum haemorrhage: an integrative review of the literature
Source: BMC Pregnancy Childbirth. 2015 Sep 28;15:230. doi: 10.1186/s12884-015-0653-6 (PMC4587838; doi:10.1186/s12884-015-0653-6)
Supplement: Additional file 2: — Characteristics of qualitative studies included after quality review. (PDF 241 kb) [file 12884_2015_653_MOESM2_ESM.pdf]

Additional File 1: Characteristics of qualitative studies included after quality review

**Author /date**	**Vaate et al (2002)**	**Sibley et al (2007)**	**Matsuyama and Moji (2008)**	**Jangsten et al (2010)**
**Code**	1	2	3	4
**Topic area and aims**	To assess knowledge, attitudes, practices, and role of trained Gambian traditional birth attendants (TBAs) in prevention, recognition and management of postpartum haemorrhage	To identify local cultural theories that women and (untrained) TBAs use to recognize and care for postpartum problems, including PPH.	To explore local understanding of bleeding during pregnancy and at delivery, and the meaning attached to it.	To explore midwives' experiences of management of the third stage of labour and assessment of risk.
**Theoretical perspective**	No clear theoretical perspective	No clear theoretical perspective	Grounded theory	No clear theoretical perspective
**Design**	Semi-structured interview study and group discussions	Qualitative data collection and multivariate analysis	Interview study and case histories	Focus group study
**Setting and context**	Rural villages in The Gambia, West Africa	Rural Bangladesh	Rural Nepal	Six hospital labour wards, Sweden
**Sampling strategy and sampling size**	22 trained TBAs and their 2 supervisors were identified in the 12 villages of interest	20 informants from 4 groups: women of reproductive age who gave birth in 2005; women between 50 and 70 years living in extended family; trained and skilled birth attendants.	Snowball sampling	Recruitment through the labour ward manager at each hospital. Criteria set by research team – e.g. labour ward midwifery experience for more than 15 years.
**Other participant characteristics**	TBAs had received 6 weeks training and attended an average of 15 births per annum. Supervisors were Government employed Community Health Nurse-Midwives and provided on-going education and supervision.	Almost all were women, parity 1-3, married, Muslim, no formal education or none beyond 5th year. 18-70 years. Women of reproductive age younger, lower parity and reported more formal education than TBAs and elder women. TBAs had attended 10 or fewer births in the previous year.	28 participants were interviewed 2-5 times each. Key informants, included village wise men, wise women, village health volunteers, mothers-in-law, women with small children, husbands, TBAs, traditional healers and pharmacists. They were carefully selected after an initial interview.	32 midwives in 6 focus groups<br>4 had worked for less than 15 years (14,11,9,and 6 years). Participants had a total of 772 years of experience (average 24 years). Ages between 24 and 65 years
**Data collection methods**	Semi-structured interviews in TBAs home for 45-60 minutes, and group discussions in a central neutral location.	Face-to-face interview using semi-structured questionnaire with open-ended questions. Free-listing also carried out.	Semi-structured interviews using ethnographic interview guides. Also used free-listing, ranking and pile sorts	Focus group discussions, tape recorded and transcribed verbatim
**Analytical approach**	Data was 'ordered' in relation to the research questions and categorised into themes	SPSS for demographic and social data. Qualitative data coded using Atlas software.	Content analysis	Content analysis
**Quality rating**	C	C	B	B

Author /date /country	Kalim et al (2009)	Elmir et al (2012)	Mapp and Hudson (2005)	
**Code**	5	6	13	
**Topic area and aims**	Explored the differences in perceived knowledge and care-seeking behaviours of women in relation to PPH or eclampsia	To describe women's experiences of having an emergency hysterectomy following a severe PPH	Women's 'lived expereince' of obstetric emergencies	
**Theoretical perspective**	No clear theoretical perspective	Naturalistic inquiry ???	Husserlian phenomenology	
**Design**	'A mix of qualitative research methods'	Interview study	Interview study	
**Setting and context**	Districts of Sylhet and Jessore, Bangladesh	Australia	NHS Hospital Trust, South West of England	
**Sampling strategy and sampling size**	Respondents chosen randomly from address lists	21 women self-selected following a media release, posters and flyers in a range of public locations.	A sample of 10 women, recruited following two local radio interviews and an advertisement in a local newpaper	
**Other participant characteristics**	Women of reproductive age (15-49 years) and elderly women (50-70 years)	Age of women: 24-57 years. Time from hysterectomy to interview was 5 weeks to 28 years. 13 women had a caesarean birth, 8 women had vaginal birth.	One woman experienced eclampsia, 5 women experienced placental abruption, 7 women experienced moderate and major postpartum haemorrhage, of which 2 led to hysterectomy.	
**Data collection methods**	Free listing, rating exercises, hypothetical case scenarios and in-depth interviews	Face-to-face, telephone and internet email interviews.	Face to face interview at a location chosen by the woman.	
**Analytical approach**	Content analysis and ranking	'Data inductively analysed'	Colaizzi's method	
**Quality rating**	B	A/B	C/D	
